# Production of Inactivated Influenza H5N1 Vaccines from MDCK Cells in Serum-Free Medium

**DOI:** 10.1371/journal.pone.0014578

**Published:** 2011-01-24

**Authors:** Alan Yung-Chih Hu, Yu-Fen Tseng, Tsai-Chuan Weng, Chien-Chun Liao, Johnson Wu, Ai-Hsiang Chou, Hsin-Ju Chao, Anna Gu, Janice Chen, Su-Chen Lin, Chia-Hsin Hsiao, Suh-Chin Wu, Pele Chong

**Affiliations:** 1 Vaccine Research and Development Center, National Health Research Institutes, Zhunan, Taiwan Authority; 2 Institute of Biotechnology, National Tsing Hua University, Hsinchu, Taiwan Authority; 3 Graduate Institute of Immunology, China Medical University, Taichung, Taiwan Authority; Saint Louis University, United States of America

## Abstract

**Background:**

Highly pathogenic influenza viruses pose a constant threat which could lead to a global pandemic. Vaccination remains the principal measure to reduce morbidity and mortality from such pandemics. The availability and surging demand for pandemic vaccines needs to be addressed in the preparedness plans. This study presents an improved high-yield manufacturing process for the inactivated influenza H5N1 vaccines using Madin-Darby canine kidney (MDCK) cells grown in a serum-free (SF) medium microcarrier cell culture system.

**Principal Finding:**

The current study has evaluated the performance of cell adaptation switched from serum-containing (SC) medium to several commercial SF media. The selected SF medium was further evaluated in various bioreactor culture systems for process scale-up evaluation. No significant difference was found in the cell growth in different sizes of bioreactors studied. In the 7.5 L bioreactor runs, the cell concentration reached to 2.3×10^6^ cells/mL after 5 days. The maximum virus titers of 1024 Hemagglutinin (HA) units/50 µL and 7.1±0.3×10^8^ pfu/mL were obtained after 3 days infection. The concentration of HA antigen as determined by SRID was found to be 14.1 µg/mL which was higher than those obtained from the SC medium. A mouse immunogenicity study showed that the formalin-inactivated purified SF vaccine candidate formulated with alum adjuvant could induce protective level of virus neutralization titers similar to those obtained from the SC medium. In addition, the H5N1 viruses produced from either SC or SF media showed the same antigenic reactivity with the NIBRG14 standard antisera.

**Conclusions:**

The advantages of this SF cell-based manufacturing process could reduce the animal serum contamination, the cost and lot-to-lot variation of SC medium production. This study provides useful information to manufacturers that are planning to use SF medium for cell-based influenza vaccine production.

## Introduction

Influenza is a highly contagious disease that affects the respiratory system, and some severe cases could lead to hospitalization or even death. In recent years, human infection with highly pathogenic avian influenza H5N1 viruses has and still poses a serious threat to public health. According to the bulletin of the World Health Organization (WHO), there were 293 deaths among the 496 human cases recorded in 15 countries throughout Africa, Asia, and Europe [1]. If H5N1 viruses continue to evolve and acquire the ability to cause widespread human-to-human transmission, this could result in an influenza pandemic. When a pandemic occurs, the outbreak will have significant impacts on health systems and economies in every affected country. The WHO believes that vaccination is the best preventive method to reduce the chance of severe illness or death when humans are exposed to H5N1 viruses. To prevent such pandemics, effective influenza vaccines should be made available as early as possible.

In the past, inactivated seasonal influenza vaccines have been manufactured by egg-based processes; however, the current global supply using this method is only able to cover a small percentage of the world's growing population. The efficiency of this manufacturing method is low, and it requires one to two eggs to produce one dose of vaccine [2]. Furthermore, the surge demand of H5N1 vaccine would require the switch from the seasonal vaccine production to pandemic vaccine manufacturing processes that currently are the bottle-neck and inadequately addressed by the vaccine manufacturers to meet the global vaccination program recommended by the WHO. An alternative to the egg-based processes is virus propagation in mammalian cell lines which has been used for the production of influenza vaccines [3], [4], [5]. Cell-derived influenza vaccines are capable of providing equivalent or even better protection in animal models than those obtained from egg-derived vaccines [6], [7]. In addition, these vaccines were found to be safe and highly efficacious in humans [2], [8], [9], [10]. Cell-based flu vaccines offer a number of advantages over the traditional method: (a) cell lines are fully characterized and in compliance with regulatory guidelines [2]; (b) the raw materials for production are defined and can be easily produced in a short period [2], [9]. There are two regulatory-approved continuous cell lines being used for influenza vaccine production: MDCK (Madin-Darby canine kidney) cells and Vero (African green monkey kidney) cells [5], [8], [10]. These two cell lines can be cultured either in free-suspension or in a microcarrier culture system.

Serum, used as the source of nutrients, hormones and growth factors, is required for optimal growth of mammalian cells [11]. These serum factors also facilitate the attachment and spreading of cells, and provide protection against mechanical damage and shear forces [12], [13]. Besides these advantages, however, serum may contain unwanted contaminants which are a primary concern in the safety of biological products [14]. In addition to the potential adventitious viral contaminants and prion contamination, SC medium also has other production issues such as lot-to-lot variability. SC medium usually contains a high percentage of serum content (up to 10%), which increases difficulty in downstream purification. The switch from SC medium to SF medium in animal cell cultures has become a major trend for the cell-based products [15], [16], [17], [18], [19]. Some influenza virus production in MDCK cells grown on microcarriers in SF medium have been reported in the literature [16], [20]. Although the cell-based seasonal flu vaccines are available in European markets, there is little information available on the manufacturing processes and the culturing systems. In addition, because of intellectual property rights and the proprietary technologies used in these vaccine products, the availability of comparison studies on virus and product yield influenced by the compositions of culture medium between SC and SF medium and systems used are very limited.

In this study, we describe a well defined manufacturing process for influenza H5N1 vaccine production that enables the switch from Dulbecco's Modified Eagle Medium (DMEM) supplemented with 5% fetal bovine serum (FBS) to SF medium (Plus-MDCK) in a microcarrier cell culture bioreactor system for MDCK cell propagation. Higher virus yield was found in the selected SF medium. In addition, the antigenicity analysis and mouse immunogenicity study have shown that MDCK cell-based vaccine produced in SF medium are equivalent to those produced in SC medium. The current results also demonstrate the production scalability from the spinner flasks to a pilot-scale microcarrier bioreactor system.

## Results

### Evaluation and selection of SF medium

**Figure 1:**
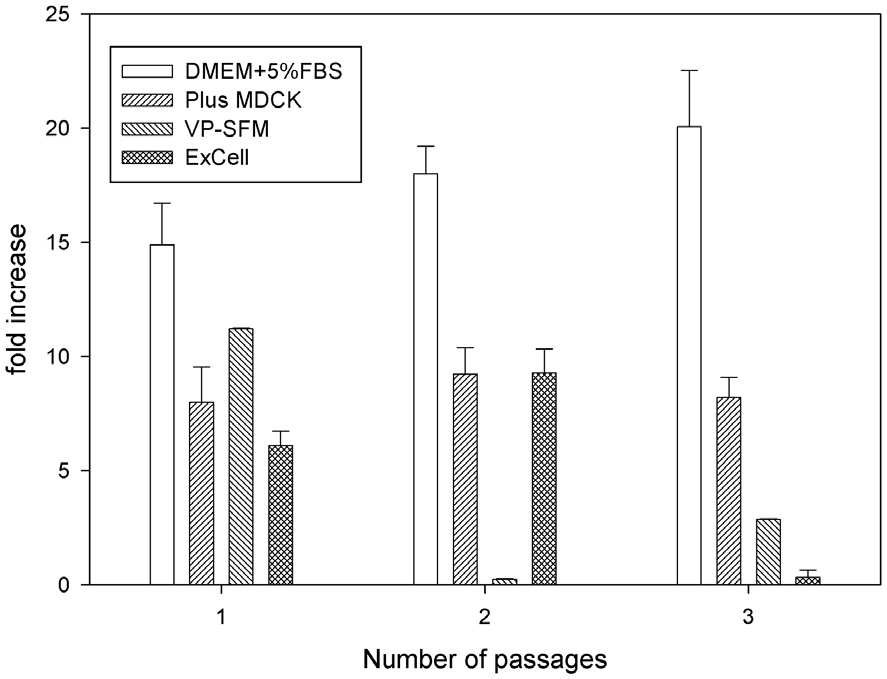
Comparison of MDCK cell growth in different culture medium. Three commercially available serum-free media (Plus-MDCK, VP-SFM, and ExCell) and one SC medium (DMEM+5% FBS) were used to evaluate the suitability for MDCK cells directly grown in 75 cm^2^ T-flasks. The thawed MDCK cells were directly cultured in four different media over 3 passages. The cell numbers were determined based on the harvest when the cell grown in SC had reached 90% confluency in day 3. The cell count in each medium was performed in triplicate. The increase of cell growth (number of fold increase) is calculated based on the final total cell number divided by the initial cell number.

To avoid the long duration for MDCK cells to adapt to a new culture media, the performance of different media in cell growth was screened and evaluated by the direct adaption method. The selection criteria were based on consistent cell growth performance over a few passages. The cells were cultured in three different commercially available SF media (Plus-MDCK, VP-SFM and ExCell) and one SC medium (DMEM with 5% FBS supplement) in 75 cm^2^ flasks over three passages after inoculation. The initial seeding cell number was 1.0×10^6^ cells/per flask. In all experiments, the final glucose concentration was maintained adequately to avoid the depletion of nutrients. Indicators such as total cell number, viability, and morphology were monitored over the testing period. The total cell numbers obtained from different media were calculated based on the cells cultured in the T-flask with DMEM+5% FBS (SC) medium reaching 90% confluency on day 3. The samples in each culture media were performed in triplicate. The definition of cell fold-increase is defined and calculated based on the final total cell number divided by the initial cell number. **Figure 1** illustrates the increase of MDCK cell growth in different culture media with 3 consecutive passages. As shown in figure 1, the cell counts in the SC medium were found to be higher than those found in the SF media (p<0.01). This could be due to serum provided containing more growth factors for MDCK cells to grow. In contrast, the SF media might not have sufficient growth factors like the SC medium for supporting cell growth. Without further culture medium adaption, VP-SFM and ExCell media showed high fluctuations and lower cell growth rates. The culture in Plus-MDCK medium with three passages showed similar levels in cell growth profile. It appears that the Plus-MDCK SF medium could consistently promote cell growth and meet the medium selection criteria. Thus, the Plus-MDCK SF medium was selected and used in all later experiments.

### Serum-free medium cultivations in various sizes of microcarrier bioreactor systems

**Figure 2:**
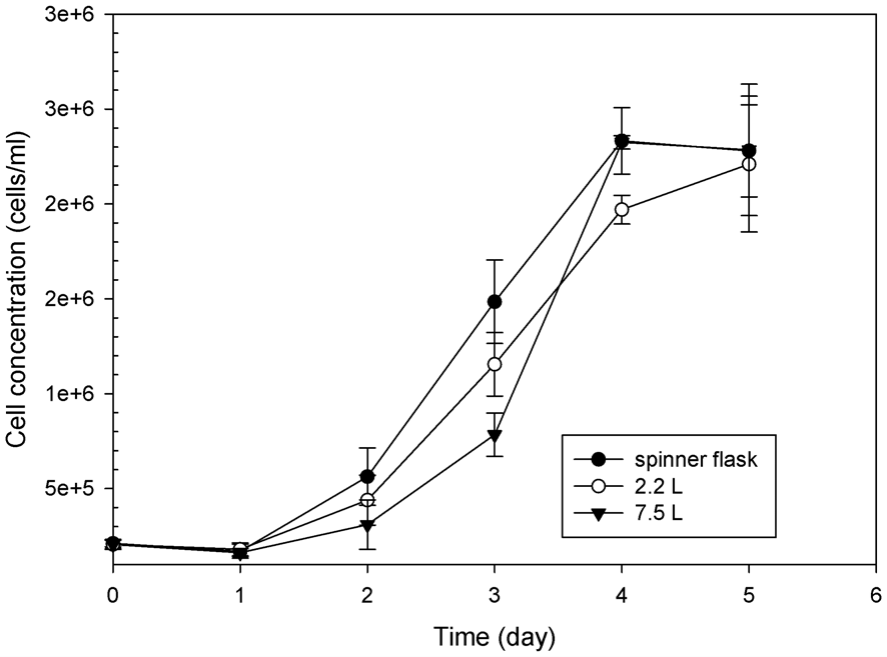
The growth of MDCK cells on microcarrier in each cultured system. MDCK cells were grown either in the 125 mL spinner flask, or 2.2 L or 7.5 L bioreactors with 5 g/L of Cytodex 1 microcarriers. Cell densities were measured on each day and found to increase more than 10-fold from an initial concentration of 2.0×10^5^ cells/ml to confluency at 2.3×10^6^ cells/ml after 5 days. The cell growth in various bioreactors did not show any significant difference of cell density based on the student t-test.

To evaluate the influences of culture-vessel size on cell growth in the Plus-MDCK medium, MDCK cells were cultured in three different-sized bioreactors with a fixed amount (5 g/L) of Cytodex 1 microcarriers. **Figure 2** shows the MDCK cell growth profiles in 125 mL spinner flask, the 2.2 L and 7.5 L bioreactors. Shortly after inoculation, the cells had attached to the microcarriers and started to colonize the surface. The cell density in three different bioreactors decreased slightly at the beginning and then increased steadily after inoculation. After 5 days inoculation, the cells on microcarriers were >90% confluent. Cell densities increased more than 10-fold from an initial concentration of 2.0×10^5^ cells/mL to 2.3×10^6^ cells/mL. During the cultivation of the cells grown in various sizes of bioreactors, no significant difference in cell density was observed.

### Production of viral antigens

The MDCK cells in the 125 mL spinner flask, the 2.2 L and 7.5 L bioreactors were infected with NIBRG14 viruses using a very low multiplicity of infection (MOI). Due to viral infection, the cytopathic effect (CPE) was observed in the MDCK cells grown on the microcarriers. It is worth pointing out that the concentrations of glucose and other substrates depleted readily after 48 hrs infection (data not shown) which was before the viral infection media exchange was performed to avoid the nutrient limitation. Virus titers (HA and plaque assay) are evaluated and shown in **Table 1**. The virus yield and HA titers were found to be 1024 HA titer/50 µL and >5×10^8^ pfu/mL and no significant differences between the various vessel sizes used (125 mL spinner flask, the 2.2 L and 7.5 L bioreactors). Therefore, all further experiments were performed with 7.5 L bioreactor.

**Table 1:** Production of virus titers in various microcarrier/bioreactor systems.

	HA titer*(HA units/50 µl)	plaque assay(pfu/ml)
Spinner flask	1024	4.8±0.3×10^8^
2.2 L bioreactor	1024	7.6±0.5×10^8^
7.5 L bioreactor	1024	7.1±0.3×10^8^

*The HA titer were performed in triplicate in 96-well microplates using turkey red blood cells according to the standard technique [29].

**Figure 3:**
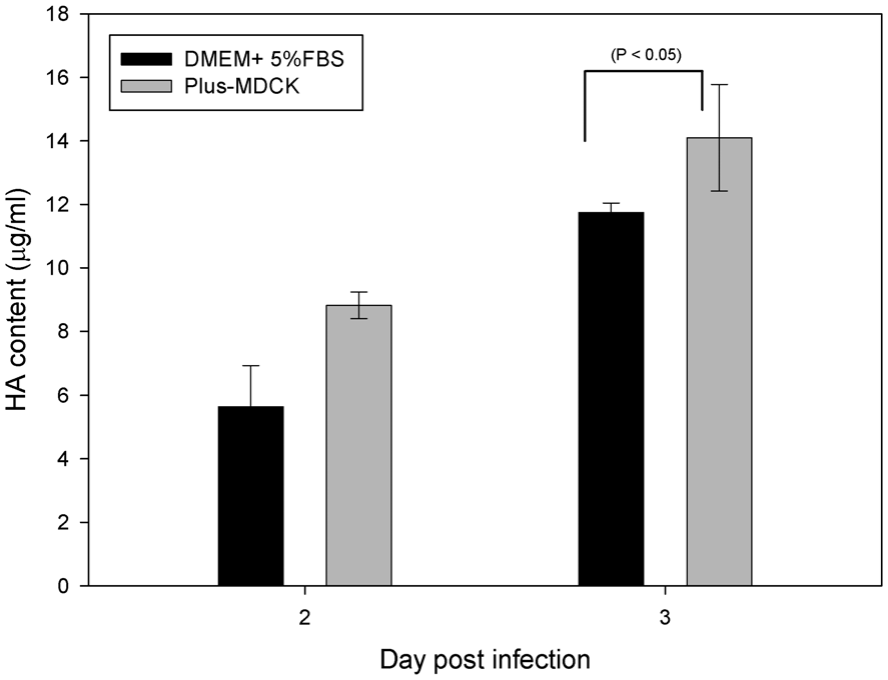
Production of HA antigens produced either in SC or SF medium. Samples taken from the 7.5 L bioreactor during the infection period were analyzed for HA antigen content. The harvest of HA antigen reached a peak at 14.1 µg/mL on day 3 post infection, as determined by SRID assay. By contrast, the harvest from the SC medium was 12.7 µg of HA/mL.

Similar to the spinner flask results, MDCK cell density grown in the 7.5 L bioreactor was found to be higher in the SC medium (>6×10^6^ cell/mL) than those obtained from the SF medium (2.3×10^6^ cell/mL). But the virus yield and HA titers at the 3^rd^ day harvest had very similar values (1024 HA titer/50 µL and >7×10^8^ pfu/mL) obtained from the SC study. Samples taken from the 7.5 L bioreactor during the infection period were further analyzed for HA antigen concentration, and then compared with the values obtained from the SC study. The HA antigen concentrations were determined by the single-radial immunediffusion (SRID) assay [21] and shown in Figure 3. The HA concentrations increased with infection time. The concentrations of HA antigen in day 1 were under the detection limit. On the harvest day, the HA antigen concentration from the SF culture was found to be 14.1 µg/mL on average over three runs, whereas the average 12.7 µg of HA antigen/mL was found in the SC culture. It shows that the titer of HA antigen produced from SF medium was slightly higher than that from SC medium (p<0.05). Influenza H5N1 virus produced in the SF medium was further concentrated, sucrose-gradient purified, and formalin-inactivated as described in the Materials and Methods section. Based on the amount of HA found in the harvest, the average of the total recovery rate of HA yield was around 45%. In the antigenicity study, the experiments were performed on the H5N1 viruses produced from different culture (SC and SF) media and found to have the same reactive titer (400) with the NIBRG14 standard antibody obtained from the WHO reference laboratory. These titers show that high-yield MDCK cell-based influenza vaccine production is possible using the current SF bioreactor technology.

### Mouse immunogenicity study

The purified H5N1 viruses were purified by sucrose gradient zonal centrifugation and then pooled from high HA-titer fractions; thus the purified samples contained only small amounts of impurities as analyzed by the Western blot using antisera raised against MDCK cell lysate (data not shown). To perform mouse immunogenicity, different amounts of the formalin-inactivated H5N1 vaccine candidate produced in the SF medium were formulated with alum adjuvant and then injected into different groups of mice to test their ability to induce immune responses. All mice were immunized with two doses. Results obtained from the control group (PBS in Group 1) and the two different doses of H5N1 flu vaccine candidates (0.2 and 1 µg of HA/dose in Groups 2 and 3, respectively) were summarized in Table 2. The GMT of HI titers generated from mice immunized with low dosage (0.2 µg of HA, Group 2) and high dosage (1 µg of HA, Group3) were found to be 80 and 285, respectively. These results indicate that the H5N1 vaccine antigens could induce dosage-dependent antibody responses after two injections. In addition, 100% of seroprotection rates (40 HI titer is considered as the level of seroprotection by CHMP criteria) were observed in both Groups 2 and 3 after two doses of immunization. The data presented in **Table 2** demonstrated that 100% protection could be achieved with as little as 0.2 µg of HA antigen in the mouse model. A similar finding was reported by a previous study [10]. The current results show that the cell-based H5N1 viral antigens produced from SF medium could induce strong and efficacious immune responses similar to those obtained from the H5N1 virus produced from the SC medium reported in our previous study [4].

**Table 2:** Immunogenicity of inactivated influenza H5N1 vaccine candidates in mice.

	Group 1	Group2	Group 3
HA (µg/dose)AlPO4 (µg of Al/dose)Number of animals	03006	0.23006	1.03006
HI GMT (95% CI)PrevaccinationPost-dose1Post-dose2	<10<10<10	<1040 (14–112)80 (50–127)	<1032 (18–57)285 (139–583)
HI seroprotectionPrevaccinationPost-dose1Post-dose2	0%0%0%	0%67% (4/6)100% (6/6)	0%50% (3/6)100% (6/6)

## Discussion

The pandemic potential of the highly pathogenic avian influenza H5N1 virus highlights the urgent need for an effective vaccine. We and others are searching for a robust and well-controlled vaccine manufacturing system that is capable of producing large amounts of vaccine antigens within a short period of time. Traditional technologies using embryonated eggs to produce the split vaccines may not fulfill all these criteria [10]. More importantly, the cell-based process is completely independent of the supply of eggs that may be endangered during a pandemic. The use of cell culture as an alternative approach for influenza virus production has gained significant interest in recent years. Several cell lines, such as MRC-5, WI-38, FRhL, Vero and MDCK, have been assessed for their ability as the production substrates for influenza viruses [22]. In recent years, commercially available cell-based influenza vaccines are commonly produced from either Vero or MDCK cells. In 2002, a vaccine from Baxter received approval for Influject®, which is a formalin-inactivated whole-virion influenza vaccine produced in Vero cells grown in SF Medium. In 2007, Novartis received approval for its product Optaflu, a trivalent MDCK cell-derived seasonal influenza vaccine produced in SF medium. Optaflu was the first commercially inactivated flu vaccine derived from MDCK cell cultures. It is important that cell-based influenza vaccine manufacturing processes using the selected cell lines can produce high virus yield and HA titers from a wide variety of influenza virus strains. Recent studies have already shown that MDCK cells are the most suitable substrate for prorogating influenza virus [2], [4], [7], [16], [20], [22]. MDCK cells not only enable the growth of different influenza virus strains but also produce good virus yield with relatively high HA titers [23]. As most vaccine manufactures have kept secret about their technical know-how and proprietary manufacturing processes, little information is known about how the commercial vaccines were made. Thus, it is not easy to make appropriate comparisons on each process step and to analyze how different medium formulations could influence the cell growth and virus yield. We describe here the advantages of an inactivated whole virus H5N1 vaccine manufacturing process using MDCK-cell based cell culture system in SF medium.

Over the past decades, serum was used as the essential component of cell-culture media for facilitating cell growth. However, the use of serum presents several disadvantages such as potential to induce hypersensitivity [24], batch variability, possibility of introducing contaminants such as bovine viruses, prions, mycoplamas, etc. Therefore, process development for biopharmaceutical products under conditions which are free of animal components reduces the contamination risk and the difficulty in removing the impurities from the downstream purification steps [17]. As a result, these products will be of high quality and consistently safe [25]. In recent years, much attention has focused on the use of SF medium instead of SC medium in cell-based vaccine and biologics production. It is a demanding task to develop a viable process using SF medium formulation for both cell growth and virus replication. Currently, there is no validated analytical method for monitoring cell or virus growth. Empirical experiments appear to be a reliable approach used to determine the optimal condition for both cell growth and virus replication stages. Several commercially available media such as VP-SFM from Invitrogen; ExCell MDCK from JRH; Plus MDCK from Cesco Bioengineering are designed for MDCK cell cultures. The current study was performed to identify a high virus-yield SF medium as a possible replacement for the SC process reported in our previous study [4]. Three commercially available SF media listed above were assessed for their ability promoting MDCK cell growth and compared with those obtained from a SC medium. In the SF medium screening experiments, the direct adaptation method for cell growth is considered to be the easiest way to minimize adaptation time switching from SC medium to SF medium. This approach can reduce the concerns of cell physiology changes due to long sub-cultured adaption. The T-flasks was used to assess whether these SF medium could support MDCK cell growth in a static environment. Cells sub-cultivated over three passages in Plus-MDCK showed a consistent performance. This is the key criteria to evaluate the feasibility of using this SF medium for further process development. Other SF media such as VP-SFM and ExCell did not support MDCK cell growth well in our direct cell adaption approach. It indicates that these two SF media are not yet optimized for MDCK cells. A similar observation was also reported by Genzel et. al. [20] that ExCell medium did not support MDCK cell growth. Although Plus-MDCK medium was shown to be a good candidate for the production of influenza H5N1 vaccines, very limited information is known about the key factors in this SF medium formulation. Thus, it is hard to evaluate how these components influence cell density and virus yield.

When cells are grown in dynamic environments such as that of bioreactor cultivation, the viability of growth of cells can be strongly affected by shear in the environment [12]. This is always a challenge to design a robust system that can support cells growing in SF medium. Cultures in serum-supplemented media protect cells against the detrimental effect of sparging [13]. This situation could be worse if cells are cultured in SF medium. The addition of shear protectant such as Pluronic F-68 in SF medium is often needed to protect cells from damage by shear forces [26]. The solution to this method is to use bubble-free aeration by using silicone tubing [27]. Batch bioreactor cultures of MDCK cells in SF medium have been reported by others [3], [20], [28]. The virus titers among these studies were not very high (∼10^6^ pfu/mL). In the current study, a bubble-free bioreactor culture of MDCK cells in Plus-MDCK medium with 5 g/L of microcarriers using perfusion mode, resulted in a higher virus titer level (∼10^8^ pfu/mL as shown in Table 1). Without a long cell adaptation in medium exchange procedure, the current results confirm that a fast and user-friendly SF medium manufacturing process has been developed and the culture conditions are established for scale-up.

A comparison of HA protein antigen produced in the SF medium to the results from the SC medium was performed. Interestingly, the cell density was lower in SF medium, but the yield of H5N1 antigen was found to be slightly higher based on the SRID assay (Figures 1 & 3). This observation was never reported in the literature. This phenomenon is unclear and not well understood. It could be the components of residual serum acting as anti-protease to neutralizing and stopping the host trypsin-like protease cleavage of HA antigens that is normally required for flu virus replication. Another explanation could be the different composition between SF and SC medium resulted in different cellular metabolism or apoptosis that could influence the virus replication and virus yield.

In summary, the current study has demonstrated that: (i) SF medium (Plus MDCK) could support both the cell-growth in the solid microcarriers and H5N1 virus-replication in the well controlled and scalable bioreactors; (ii) the cell-based flu vaccines could be manufactured in a safe and user-friendly process with fewer contaminants and consistently high yield. The use of SF medium certainly could help to reduce the concerns of the bovine spongiform encephalopathy (BSE) issue, lot-to-lot variation and simplifying raw-material sources analysis. Plus-MDCK medium not only supported MDCK cells directly, which adapted to grow well in both T-flasks and bioreactors, but also enhanced the virus-replication and generated high infectious virus titers (∼10^8^ pfu/mL) after 72 h following infection. In terms of material cost, we found Plus-MDCK medium to be 55% less expensive than SC medium. This work further illustrates that the microcarrier system using Plus-MDCK medium poses an alternative approach for the production of influenza H5N1 viruses at high yields. Current preliminary studies at the 30-L scale using this approach have been very successful (data not shown). Further work will be extended to perform studies with the pilot-scale 150 L bioreactor as well as other single-use culture systems.

## Materials and Methods

### Ethics Statement

All experiments were conducted in accordance with the guidelines of Laboratory Animal Center of NHRI. The animal use protocols have been reviewed and approved by the NHRI Institutional Animal Care and Use Committee (Approved protocol no. NHRI-095054-A).

### Virus and cells

The origin of the NIBRG-14 (derived from A/Vietnam/1194/2004) vaccine strain and MDCK cells are described in our previous study [4]. The viruses were further amplified to generate virus stocks in MDCK cells. MDCK cells were grown in SF medium (Plus-MDCK). Master and working cell banks cultured were prepared using SC medium, and then further characterized to fulfill the cGMP guidelines for manufacturing biological products.

### Medium selection in 75 cm^2^ T-flasks

Three commercially available SF media (Plus-MDCK, VP-SFM and ExCell) and one SC medium were used in the medium selection study. The SC medium contained basal medium DMEM (Invitrogen, UK) and 5% fetal bovine serum (FBS). The FBS was purchased from Moregate Biotech (Australia). Plus-MDCK medium was purchased from Cesco Bioengineering Co., Taiwan. VP-SFM (cat. no. 11681) was supplied by Invitrogen (UK). VP-SFM and ExCell (cat. no. 14581C) was supplemented with 4 mM L-glutamine before use. Each 75 cm^2^ T-flask was inoculated with approximately 1.0×10^6^ cells and grown for 3 days in 20 mL medium. Each culture medium was performed in triplicate. When cell numbers in the T-flask with DMEM+5% FBS medium reached 90% confluency (approximately three days after inoculation), the cells in each T-flask were detached by Trypsin-EDTA (Invitrogen, UK) for cell counting. After counting cells, the same seeding cell number of 1.0×10^6^ cells was sub-cultured to new flasks. The procedure was repeated in triplicate.

### Cell growth in spinner flasks

Cell culture was carried out in 125 mL spinner flasks (Corning, USA) containing 100 mL of culture medium at 37°C in a 5% CO_2_ incubator. Cells were cultivated in Plus MDCK. The stirring speed was maintained at 45 rpm. The spinner flasks were inoculated with 2×10^5^ cells/mL. The experiments were carried out in duplicate. Samples were taken daily to perform various off-line analyses.

### Bioreactor cultures

The cultures were performed in 2.2 L and 7.5 L bioreactors (NBS, USA) with working volumes of 1.4 L and 5 L, respectively. Cytodex 1 microcarriers (GE Healthcare, USA) were hydrated, autoclaved, and preconditioned according to the manufacturer's instructions before use. The seeding density was with 2.0×10^5^ cells/mL; and an agitation speed was maintained at 35–50 rpm. During cell growth, pH, dissolved oxygen and temperature were maintained at 7, 50% air-saturation, and 37°C, respectively. Perfusion rate was adjusted daily to maintain glucose concentration at around 1 g/L. When the cells on the microcarrier were fully confluent, the medium was exchanged with 90% of fresh medium. 2 µm/mL of TPCK-trypsin was added to the medium before viral replication (Invitrogen, USA), and the cells were infected with a multiplicity of infection (MOI) of 0.00001. Samples were taken daily to determine cell density and viral assays. Each bioreactor culture was repeated twice.

### Virus titration

HA titration was conducted in 96-well microplates using turkey red blood cells (RBC) according to the standard technique [29]. Virus infectious titers were measured using plaque assay based on plaque forming units (pfu) in MDCK cells [29]. A positive control with pre-specified acceptable ranges of titers was included for conducting HA and plaque assays.

### Purification of vaccine antigens

The SF-produced H5N1 virus was purified according to the procedure described in the previous study [4]. Vaccine antigens produced from the 7.5 L bioreactor were harvested through a 0.65 µm depth filter; and the clarified solution was then concentrated by an ultrafiltration step using 300K membrane (Sartorius, Germany). The concentrated samples were purified using the sucrose-density gradient zonal centrifugation (CP-80, Hitachi, Japan). The fractions containing the purified whole virus particles were pooled together. A diafiltration step was used to remove sucrose content. The purified viruses were inactivated with 0.01% of formalin. Virus inactivation was confirmed by plaque assays. The HA antigen protein concentrations were measured using SRID assay [21]. The purified HA antigen of the SC culture from the previous study [4] was stored at −80°C and was taken for comparison study. The analysis of HA antigen was repeated three times. The standard HA antigen and antiserum were purchased from the HPA, UK.

### Antigenicity Analysis

Antigenicity analysis of the influenza viruses (NIBRG14) was carried out by HI assay using the standard anti-H5N1 antibody that was purchased from the National Institute for Biological Standards and Control (NIBSC code: 04/214). The MDCK cells were cultivated in the SC medium (DMEM+5% FBS) or the SF medium (Plus-MDCK). The same operating procedure was followed by the earlier section of cell growth in spinner flasks. The samples were taken on day 3 from 125-mL spinner flask cultures using the SC medium or the SF medium. The HI assay started at a serum dilution of 1∶100. Each sample was performed in triplicate.

### Mouse immunogenicity study

Six- to eight-week-old female BALB/c mice were immunized intramuscularly with two doses of vaccine antigen at a 2-week interval. Three groups with the same amount of adjuvant but containing different HA dosages were compared. These groups were 300 µg AlPO_4_, 0.2 µg of HA+300 µg AlPO_4_, and 1.0 µg of HA+300 µg AlPO_4_. Sera were collected on day 0, day 14, and day 21 for measuring HI titers. The animal study was approved by the NHRI Institutional Animal Care and Use Committee (Approved protocol no. NHRI-095054-A).

### Serological assays

Serum HI antibody titers were measured using turkey red blood cells and HA units of virus antigens following WHO's standard procedures [29]. Serum neutralizing antibody titers were determined using MDCK cells and are expressed as the reciprocal of the highest dilution of serum that gave 50% neutralization of 100 TCID_50_ of the vaccine virus following the WHO standard procedures [29]. Sera giving a negative signal in the first dilution (<1∶10) were assigned a nominal HI score of 1∶5. HI titers are expressed as the reciprocal of serum dilution. Animal cells with a serum HI titer of ≥40 were considered seroprotected according to the European Union Committee for Medicinal Products for Human Use (CHMP) criteria. The definition of serocoversion followed CHMP criteria (4-fold increase from the baseline). Seroconversion, seroprotection, geometric mean titers (GMT), and their respective 95% confidence intervals (95% CI) were applied for analyzing antibody titers.
